# A New Family of Macrocyclic Polyamino Biphenolic Ligands: Acid-Base Study, Zn(II) Coordination and Glyphosate/AMPA Binding

**DOI:** 10.3390/molecules28052031

**Published:** 2023-02-21

**Authors:** Gina Elena Giacomazzo, Daniele Paderni, Luca Giorgi, Mauro Formica, Lorenzo Mari, Riccardo Montis, Luca Conti, Eleonora Macedi, Barbara Valtancoli, Claudia Giorgi, Vieri Fusi

**Affiliations:** 1 Department of Chemistry “Ugo Schiff”, University of Florence, Via della Lastruccia 3, 50019 Sesto Fiorentino, Italy; 2 Department of Pure and Applied Sciences, University of Urbino “Carlo Bo”, Via della Stazione 4, 61029 Urbino, Italy

**Keywords:** glyphosate, AMPA, Zn(II)-complexes, polyamines, macrocycles, anion recognition

## Abstract

In this study, the ligands 23,24-dihydroxy-3,6,9,12-tetraazatricyclo[17.3.1.1(14,18)]eicosatetra-1(23),14,16,18(24),19,21-hexaene, **L1**, and 26,27-dihidroxy-3,6,9,12,15-pentaazatricyclo[20.3.1.1(17,21)]eicosaepta-1(26),17,19,21(27),22,24-hexaene, **L2**, were synthesized: they represent a new class of molecules containing a biphenol unit inserted into a macrocyclic polyamine fragment. The previously synthesized **L2** is obtained herein with a more advantageous procedure. The acid-base and Zn(II)-binding properties of **L1** and **L2** were investigated through potentiometric, UV-Vis, and fluorescence studies, revealing their possible use as chemosensors of H^+^ and Zn(II). The new peculiar design of **L1** and **L2** afforded the formation in an aqueous solution of stable Zn(II) mono (LogK 12.14 and 12.98 for **L1** and **L2**, respectively) and dinuclear (LogK 10.16 for **L2**) complexes, which can be in turn exploited as metallo-receptors for the binding of external guests, such as the popular herbicide glyphosate (*N*-(phosphonomethyl)glycine, PMG) and its primary metabolite, the aminomethylphosphonic acid (AMPA). Potentiometric studies revealed that PMG forms more stable complexes than AMPA with both **L1**- and **L2**-Zn(II) complexes, moreover PMG showed higher affinity for **L2** than for **L1**. Fluorescence studies showed instead that the **L1**-Zn(II) complex could signal the presence of AMPA through a partial quenching of the fluorescence emission. These studies unveiled therefore the utility of polyamino-phenolic ligands in the design of promising metallo-receptors for elusive environmental targets.

## 1. Introduction

*N*-(phosphonomethyl)glycine (glyphosate or PMG) is one of the most frequently used broad-spectrum organophosphorus herbicides worldwide [1,2]. Its popularity mainly derives from its effectiveness in removing unwanted weeds in pre-harvest treatments and in non-crop areas, which can be attributed to its capacity to inhibit the activity of the 5-enol-pyruvyl-shikimate-3-phosphate synthase (EPSPS) enzymes, impairing the biosynthesis of essential amino acids for the plant growth [3]. Several additional features, such as low perceived toxicity, rapid absorption by plants, and slow evolution of PMG-resistant weeds have then contributed to further extending its use.

Since its first introduction into the market in 1974, as an active ingredient of Monsanto’s herbicide Roundup^®^ [4], the global sales volume of glyphosate has increased dramatically, with over 1.6 billion kilograms of PMG that have been used only in the US in 2016 [5]. This has led to the accumulation of PMG, along with its primary metabolite aminomethylphosphonic acid (AMPA), in different environmental matrices, such as top-soil layers [6] and surface water [7], as well as in various harvest/food products [8], raising serious concerns about its use (abuse) and regulation.

Actually, there is an ongoing scientific and social controversy regarding the potential risks for the ecosystems and in particular for human health [9,10]. Indeed, contrary to the US Environmental Protection Agency (EPA), which stated that “there are no risks to public health when glyphosate is used in accordance with its current label”, other countries and international agencies reached diametrically opposed conclusions. The International Agency for Research on Cancer (IARC) classified PMG as probably carcinogenic to humans (Group 2A) [11], and several studies reported on the possible acute and chronic biological effects associable with PMG, such as cytotoxicity, carcinogenicity, teratogenicity, endocrine disruption and metabolic alterations [12,13,14,15]. In 2017, the European Union (EU) controversially voted to re-license glyphosate use for a limited period of five years and a recent extension, until December 2023, was granted to allow the European Food Safety Authority (EFSA) to conclude its examination on this timely and debated issue. Analogously, raising concerns about the chronic toxicity associated with AMPA has led to its inclusion with PMG in pollution monitoring programs [16].

It is therefore of utmost importance to develop effective methods to monitor and detect PMG and AMPA in environmental samples. Apart from some recent examples of biosensors [17,18] and SERS-based sensors [19,20], the majority of the techniques currently employed still rely on liquid-gas chromatography, capillary electrophoresis, and mass spectrometry analysis [21,22,23]. However, the zwitterionic nature, high polarity, and low molecular weights of the targeted analytes make difficult both their extraction from samples and their retention on chromatographic phases. As a consequence, preliminary time-consuming derivatization procedures and the optimization of a high number of parameters (temperature, reaction time, laboratory handling time, etc.) are usually required [24,25,26], making important the research of suitable alternatives for PMG and AMPA recognition [27].

In this respect, fluorescent chemosensors, e.g., molecules composed of a binding unit linked through a spacer to a fluorogenic unit, hold great promise in the design of novel and effective systems for anion recognition and sensing [28,29,30]. Indeed, the structural features of the spacer, the binding, and the signaling unit can be finely tuned to realize low-cost and versatile systems with increased sensitivity and selectivity. Concerning the choice of binding units, polyamine scaffolds represent optimal solutions. Indeed, besides their typically high water solubility, a fundamental pre-requisite for the application in real matrices, the high number of positively charged polyammonium groups formed in solution allows for the efficient interaction with anionic guests, stabilized via hydrogen bonding and charge-charge interactions [31,32]. Moreover, these frameworks can bind metal ions [33,34,35,36,37], affording promising metallo-receptors for anionic targets [38,39]. More in particular, a number of Zn(II) coordination compounds have been shown to possess promising features as receptors for anions of environmental and biological importance, mainly thanks to the presence of coordinatively unsaturated metal centers which can in turn act as suitable anchoring sites for the coordination of anionic guests [40,41,42]. With this regard, the use of macrocyclic ligands could ensure both selectivity towards a specific metal cation and the formation of stable complexes, featuring high binding constants [35,36,43].

Of particular interest is the coupling of polyamine-based receptor units with phenolic groups as signaling moieties [37,43,44], in the realization of versatile and effective polyamino-phenolic receptors for anionic targets. Indeed, the phenolic functions confer to these systems peculiar photochemical behaviors, making it possible to detect a selected substrate via an optical response, namely by fluorescence signaling [44,45], but they can be also involved, as phenolates, in metal-coordination, affording efficient metallo-receptors for anionic guests [38,46,47].

This scenario prompted us to recently consider the class of polyamino-phenolic ligands, in their Zn(II)-receptor forms, for the recognition and sensing of PMG and AMPA [48]. We showed that the coordination of these analytes by open chain diethylentriamine (dien) frameworks spaced by a phenol or biphenol moiety was remarkably strengthened when Zn(II) ions are present in the binding pockets of ligands. Metal ions not only had a great impact on the anion binding properties of receptors but also affected their behavior as fluorescent chemosensors. Importantly, if compared to the metal-free ligands, the metallo-receptors exhibited stricter binding selectivity patterns, imparted by the combination between the structural features of the metallo-receptors and the coordination requirements of their Zn(II) centers.

**Figure 1:**
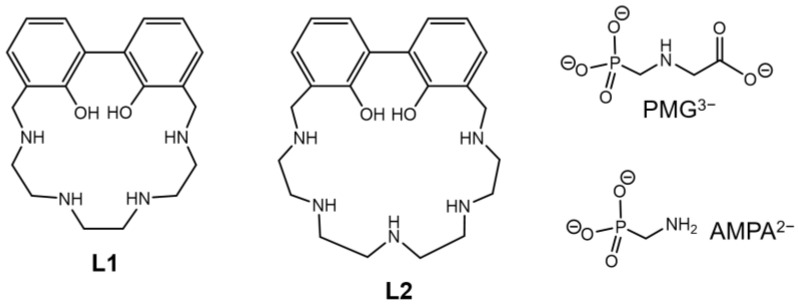
Polyamino-biphenolic ligands of this work and anionic substrates herein investigated.

In an effort to further explore and optimize the ability of this class of compounds as possible receptors for PMG and AMPA, we inserted herein the fluorogenic 1,1′-bis(2-phenol) group (BPH) into two differently sized macrocyclic frameworks, to give the two ligands 23,24-dihydroxy-3,6,9,12-tetraazatricyclo[17.3.1.1(14,18)]eicosatetra-1(23),14,16,18(24),19,21-hexaene, **L1**, and 26,27-dihidroxy-3,6,9,12,15-pentaazatricyclo[20.3.1.1(17,21)]eicosaepta-1(26),17,19,21(27),22,24-hexaene, **L2** (Figure 1). The two macrocyclic structures bear four (**L1**) or five (**L2**) nitrogen donor atoms spaced by ethylene linkers. Following the synthesis of the compounds, which, in case of **L2**, was accomplished by adopting a new procedure, the acid-base and Zn(II)-binding properties of the ligands were investigated by means of potentiometric, UV-Vis and fluorescence measurements. The same techniques were then used to inspect the ability of the Zn(II) complexes of **L1** and **L2** to bind and sense the presence of elusive anions such as PMG and AMPA in aqueous solution.

## 2. Results and Discussion

### 2.1. Synthesis

**Scheme 1:**
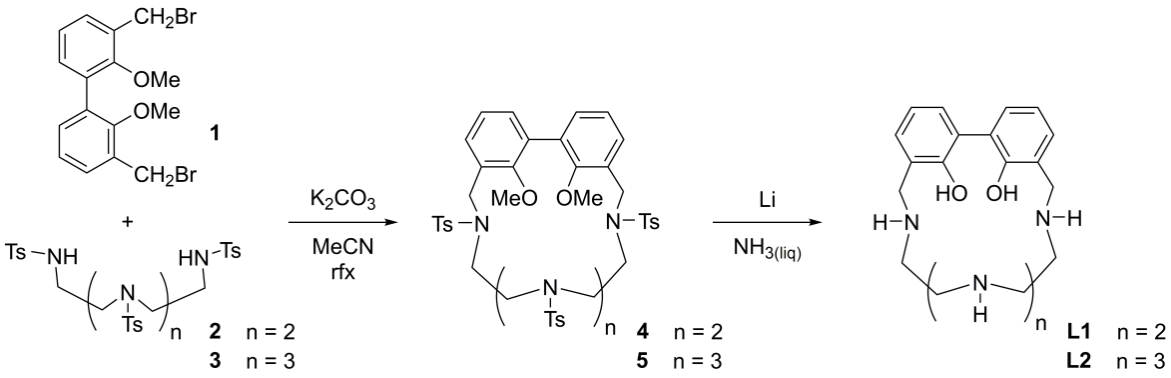
Synthetic pathway of ligands **L1** and **L2**. (n refers to the number of -CH_2_-N(Ts)-CH_2_- units in the macrocycle).

The synthetic pathway used to obtain the ligands **L1** and **L2** is reported in Scheme 1. The tosylated, phenol methyl-protected macrocycles **4** and **5** were obtained by a modification of the Richman-Atkins method, involving the cyclization of the poly-tosylated polyamines **2** and **3**, respectively, with one equivalent of the 3,3′-bis(bromomethyl)-2,2′-dimethoxybiphenyl (**1**), in the presence of the alkaline carbonate base K_2_CO_3_. The final compounds were obtained by deprotection of the macrocycles **4** and **5**. The cleavage reactions of the tosyl groups were carried out with lithium in liquid ammonia. The reducing conditions of the treatment also led to the demethylation reaction of the two ethereal methyl groups on the biphenol moiety, obtaining, after the described work-up, the ligands **L1** and **L2**. Both compounds were further purified as hydrochloride salts.

In the present paper, an alternative synthetic pathway to obtain **L2** was employed compared to the previously reported procedure [49]. This consisted of a template synthesis involving the use of toxic cadmium(II) ion as templating agent. Although the present synthesis returned a little bit lower overall yield with respect to the previous one, it allowed for avoiding the use of a toxic metal ion and was therefore preferred.

### 2.2. Protonation and Zn(II) Binding by ***L1*** and ***L2***

#### 2.2.1. Basicity of **L1** and **L2**

The acid-base properties of **L1** and **L2** were investigated by potentiometric and UV-Vis absorption and fluorescence emission measurements.

**Figure 2:**
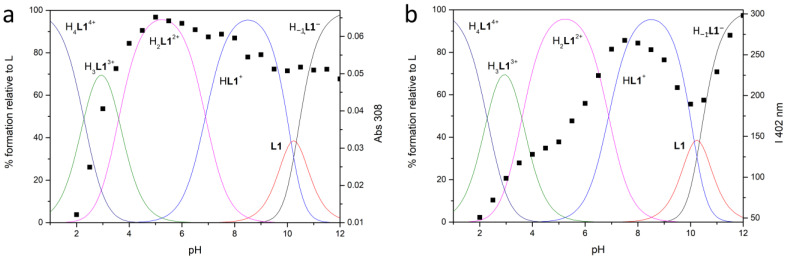
Distribution diagrams of the species of **L1** (—) in aqueous solution at 298 ± 0.1 K in 0.1 M NMe_4_Cl ([**L1**] = 1 × 10^−3^ M) and (**a**) absorption titration at λ = 308 nm and (**b**) fluorescent emission titration (■) (λ_ex_ = 288, λ_em_ = 402 nm) ([**L1**] = 1 × 10^−5^ M).

**Figure 3:**
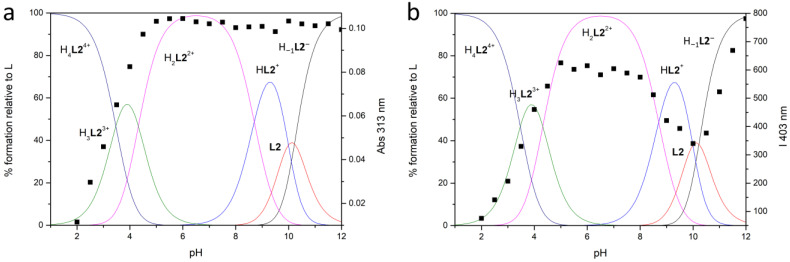
Distribution diagrams of the species of **L2** (—) in aqueous solution at 298 ± 0.1 K in 0.1 M NMe_4_Cl ([**L2**] = 1 × 10^−3^ M) and (**a**) absorption titration at λ = 313 nm and (**b**) fluorescent emission titration (■) (λ_ex_ = 288, λ_em_ = 403 nm) ([**L2**] = 1 × 10^−5^ M).

The protonation constants of **L1** and **L2** were potentiometrically determined in 0.1 M NMe_4_Cl aqueous solution at 298.1 K and are reported in Table 1 as LogK values. The distribution diagrams of the species for **L1** and **L2** are reported in Figure 2 and Figure 3, respectively.

Both neutral **L** species behave as tetraprotic bases and as monoprotic acids in these experimental conditions. The monoanionic H_−1_**L**^−^ species are forming at alkaline pH values (Figure 2 and Figure 3), suggesting that only one acidic hydrogen atom of the BPH unit can be lost under these conditions, as previously reported for similar systems [47]. Moreover, only **L1**, contrarily to **L2**, can be fully protonated in the analyzed pH range, as observed for analogous compounds bearing a phenol instead of a BPH group [43].

The high value of the first protonation constant for both ligands suggests that the H_−1_**L**^−^ and **L** species behave as strong bases in the addition of the first two protons (LogK ranging between 10.34 and 9.99); a drop in the LogK values is observed thereafter. This trend could be rationalized in terms of minimization of the electrostatic repulsions, suggesting that the first two protons locate on sites placed at a certain distance in the macrocycles, then the following protons additions occur on positions close to already protonated sites [50].

In the case of **L2**, a greater grouping of the LogK values for the addition reactions of the first three protons is observed along with LogK values often higher than for **L1**, in accordance with the greater macrocyclic dimensions and number of protonatable sites of **L2**, which allow a better minimization of the electrostatic repulsion.

**Figure 4:**
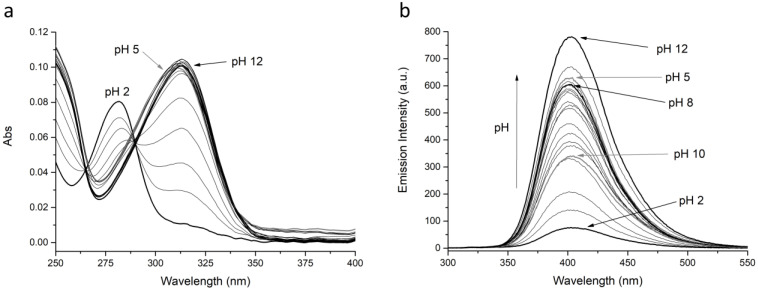
(**a**) Absorption and (**b**) fluorescence spectra of **L2** at different pH values. [**L2**] = 1 × 10^−5^ M, λ_ex_ = 288, λ_em_ = 403 nm.

Spectrophotometric UV-Vis absorption and fluorescence spectra of **L1** and **L2** were recorded in aqueous solution as a function of pH (Figure S1 and Figure 4) to get insights about the role of BPH in the fluorescence behavior and its dependence on pH. The BPH group shows indeed fluorescence properties depending on its protonation degree, moving from the least emitting neutral species to the most emitting monodeprotonated species. Usually, the fully deprotonated BPH could not be obtained unless at very high pH values [51,52,53,54,55].

The trend of the absorbance and fluorescence emission intensity at selected wavelengths (■) vs. pH, together with the distribution curves of the species for the two ligands, are reported in Figure 2 and Figure 3 for **L1** and **L2**, respectively.

At acidic pH values (pH = 2), where the protonated H_4_**L**^4+^ species are mainly present in solution, both ligands are very low emissive: the observed low intensity band is attributed to the BPH fluorophore in its neutral form, as can be inferred from the absorption spectrum (band with λ_max_ at 281 nm, see Figure S1 and Figure 4). By increasing the pH, whereas the emission wavelength remains substantially constant, the emission intensity also grows up; this can be attributed to the deprotonation of one phenolic function of the BPH group. The suggested BPH deprotonation is confirmed by the absorption spectra, where the band at 281 nm, by moving towards higher pH values, is gradually replaced by a band with λ_max_ at 308 or 313 nm for **L1** and **L2**, respectively (Figure S1 and Figure 4), ascribable to the deprotonated BPH group. Therefore, as in similar systems, the fluorescence emission of both **L1** and **L2** depends on the protonation degree of the BPH fluorophore. However, contrarily to similar but open chain systems [45,47], where the emission stays low and constant up to pH 5, in the present cases the emission intensity starts increasing at pH > 2.

More in details, in the case of **L2**, the emission increase at pH > 2 goes along with the appearance of the H_3_**L2**^3+^ species and reaches a relative maximum at pH 5, where H_2_**L2**^2+^ is prevalent in solution. The emission remains constant as long as H_2_**L2**^2+^ is prevalent in solution (5 ≤ pH ≤ 8), then a moderate drop is observed with the appearance of the H**L2**^+^ species. A relative minimum in the emission intensity is observed when the neutral **L2** species is completely formed, then the intensity steeply increases again at pH > 10, reaching the maximum at pH 12, ascribable to the formation of the anionic H_−1_**L2**^−^ species.

In the case of **L1** a similar behavior can be described, the emission intensity increasing with pH from 2 to 7.5 (H_3_**L1**^3+^ → H**L1**^+^), then decreasing up to pH 10 (neutral **L1** species), rising again to reach the maximum emission at pH 12, together with the formation of the H_−1_**L1**^−^ species.

The observed fluorescence trend for **L2** could be rationalized in terms of deprotonation of the BPH fluorophore, to give the H_3_**L2**^3+^ species, along with the formation of O^…^HN and/or O^…^HO hydrogen bonds, that could either negatively or positively impact the fluorescence emission. Indeed, the formation of an intramolecular H-bond between the two oxygen functions of BPH, that stabilizes the hydrogen atom in the monoanionic BPH, is known to rise the fluorescence emission, due to the increase of the co-planarity and the rigidity of the biphenyl system; on the contrary, the H-bond formation between the BPH oxygen and close amine functions could decrease the emission through a nonradiative relaxation process of the excited species, due to the loss of co-planarity between the two aromatic rings [51,52,53,54,55]. Therefore, it can be suggested that in both H_2_**L2**^2+^ and H_-1_**L2**^−^ species, that feature a maximum of emission, the hydrogen atom of the monoanionic BPH group is only stabilized by an H-bond between the two oxygen functions and no other H-bonds are present.

Finally, for both ligands, the absence of fluorescence changes at the highest pH value tested suggest that even in these systems, as in previous ones, the full deprotonation of the BPH group could not be achieved under the present experimental conditions.

#### 2.2.2. Coordination of Zn(II) by **L1** and **L2**

The coordination behavior of **L1** and **L2** towards Zn(II) was investigated by potentiometric, UV-Vis absorption and fluorescence emission measurements.

The stability constants for the equilibrium reactions were potentiometrically determined in 0.1 M NMe_4_Cl aqueous solution at 298.1 K and are reported in Table 2.

**Figure 5:**
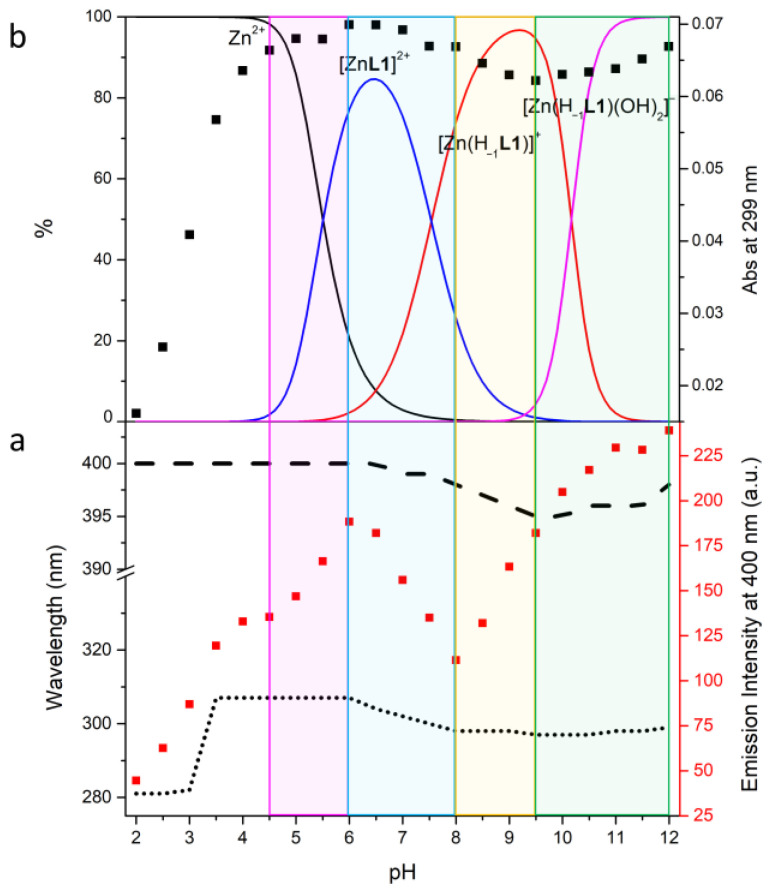
Fluorescence emission titration (λ_ex_ = 288 nm) (■), absorption wavelength trend (**^…^**), emission wavelength trend (---) (**a**); absorption titration at λ = 299 nm (■), distribution diagrams of the species (−) for the Zn(II)/**L1** system as a function of pH in aqueous solution (**b**): *I* = 0.1 M NMe_4_Cl at 298.1 ± 0.1 K, [**L1**] = 1 × 10^−3^ M, [Zn^2+^] = 1 × 10^−3^ M.

**Figure 6:**
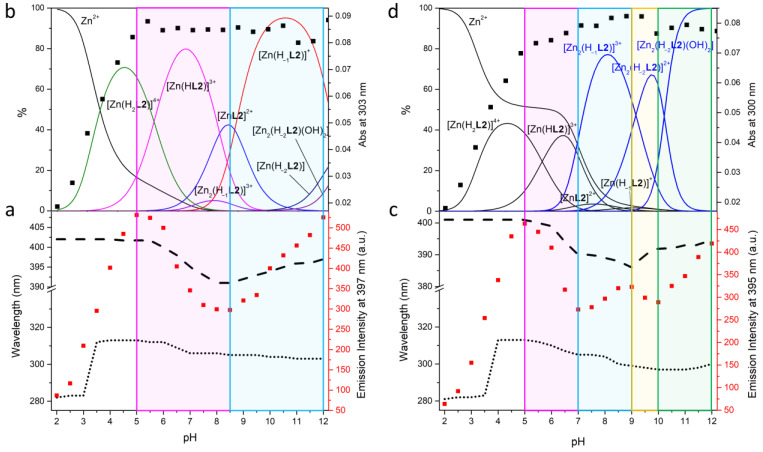
Fluorescence emission titration (λ_ex_ = 288 nm) (■), absorption wavelength trend (**^…^**), emission wavelength trend (---) (**a**); absorption titration at λ = 303 nm (**L2**: Zn(II) 1:1, (**b**) and 300 nm (**L2**: Zn(II) 1:2, (**d**) (■), distribution diagrams of the species (−) for the Zn(II)/**L2** system as a function of pH in aqueous solution (**c**): *I* = 0.1 M NMe_4_Cl at 298.1 ± 0.1 K, [**L1**] = 1 × 10^−3^ M, [Zn^2+^] = 1 × 10^−3^ M (left), [Zn^2+^] = 2 × 10^−3^ M (right).

Both ligands are able to form mononuclear species with Zn(II) ions, while only **L2** can form dinuclear complexes with the metal ion. When the **L2**/Zn(II) molar ratio is 1:1, the mononuclear species prevail in solution in the whole tested pH range; on the contrary, when the **L2**/Zn(II) molar ratio is 1:2, the dinuclear species are mainly present in solution at pH > 6. The distribution diagrams of the species for the **L1**/Zn(II) (1:1 molar ratio) and **L2**/Zn(II) (1:1 and 1:2 molar ratios) systems as a function of pH are reported in Figure 5 and Figure 6.

By comparing the mononuclear complexes of the two ligands having the same stoichiometry, **L2** forms more stable species than **L1** (Table 2); this can be explained by the higher number of donor atoms in **L2** than in **L1**.

For both ligands, the values for the addition constants (LogK) of Zn(II) to the H_−1_**L**^−^ species are not so different from those for the addition to the **L** species, suggesting a similar coordination environment for Zn(II) in the two species and the involvement in the first protonation step of an amine function non-coordinated to the metal center. Moreover, while the [Zn(H_−1_**L1**)]^+^ mononuclear complex can only add one hydrogen ion, the [Zn(H_−1_**L2**)]^+^ mononuclear complex can add up to three hydrogen ions; the LogK values for the addition of the first two protons to [Zn(H_−1_**L2**)]^+^ are similar to each other and to the third protonation step of the free ligand (LogK = 8.70), suggesting that such protonation processes involve nitrogen atoms not engaged in the coordination. This means that the Zn(II) ion in the mononuclear [Zn(H_−1_**L**)]^+^ complexes is probably coordinated by three amine functions of the macrocycles, besides probably only one oxygen atom of the monoanionic BPH.

Finally, both [Zn(H_−1_**L**)]^+^ complexes seem to be able to bind hydroxide species, suggesting that they are prone to add external groups. However, an alternative process such as a further deprotonation of the BPH moiety could not be ruled out for both ligands.

**L2** also forms dinuclear Zn(II) species; the LogK value for the addition of the second Zn(II) ion to [Zn(H_−1_**L2**)]^+^ is much lower than that for the first one (5.49 vs. 12.98), which can be rationalized both in terms of addition of a cation to a positively charged species and of a lower number of binding sites available in [Zn(H_−1_**L2**)]^+^ with respect to H_−1_**L2**^−^ species. Above pH 6 only dinuclear species are present in solution, each one existing in a narrow range of pH. No further protons can be added to the [Zn_2_(H_−1_**L2**)]^3+^ species, suggesting the involvement of all available donor atoms in the coordination of the two metal centers. The species is however able to bind further hydroxide groups, that probably contribute to saturate the coordination requirement of the two Zn(II) ions, making this species a suitable metallo-receptor for external ligands. However, even in this case, an alternative full deprotonation of BPH cannot be totally ruled out.

To try to understand the degree of protonation of the BPH group in the complexes and its role in the emission properties, UV-Vis and fluorescence experiments at different pH values were performed. The fluorescence spectra of **L1** + 1 equiv. of Zn(II), **L2** + 1 equiv. of Zn(II) and **L2** + 2 equiv. of Zn(II) (λ_ex_ 288 nm) recorded in aqueous solution in the pH range 2–12 are reported in Figures S2–S4. The trend of the fluorescence emission intensity (■) as a function of pH, together with the maximum absorption (**^…^**) and emission (---) wavelength is reported in Figure 5a for **L1** and Figure 6a,c for **L2**. In Figure 5b and Figure 6b,d is depicted the trend of the absorption titration at λ = 299 nm (**L1**) and λ = 303 nm (**L2**: Zn(II) 1:1) and 300 nm (**L2**: Zn(II) 1:2) (■) along with the distribution curves for the species of the two ligands.

In the case of **L1** the formation of the [Zn**L1**]^2+^ species is accompanied by a fluorescence emission increase, due to both the coordination of Zn(II) (chelation enhanced fluorescence (CHEF) effect) and the deprotonation of BPH, as confirmed by the absorption increase of the phenolate band (Figure 5b).

The deprotonation of [Zn**L1**]^2+^ to give [Zn(H_−1_**L1**)]^+^ goes along with an emission decrease and a blue shift of the emission wavelength from 400 to 395 nm. A rearrangement of the species leading to a lesser conjugation of the BPH group could explain this behavior, in agreement with the small variation of the absorption wavelength from 307 to 298 nm.

Finally, the addition of two hydroxide groups to give the [Zn(H_−1_**L1**)(OH)_2_]^−^ species probably increases the rigidity of the BPH-Zn(II) system: this could be due to the removal of the possible coordinated water molecules that would favor the vibrational dissipation of energy, thus inducing a fluorescence emission increase. However, a full deprotonation of BPH cannot be totally ruled out, as already suggested above.

In the case of **L2** in the presence of an equimolar amount of Zn(II) (Figure 6a,b), the formation of the [Zn(H_2_**L2**)]^4+^ species comes with the increase of fluorescence emission at 402 nm, that is due to both Zn(II) coordination and deprotonation of the BPH group, as highlighted by the increase of absorbance and a change of the absorption wavelength from 281 to 313 nm. The deprotonation of [Zn(H_2_**L2**)]^4+^ to give [Zn(H**L2**)]^3+^, first, and then [Zn**L2**]^2+^, induces a drop of both the fluorescence emission intensity and wavelength, from 402 to 391 nm; also, the absorption wavelength blue shifts from 313 to 305 nm. A further increase of both the fluorescence emission intensity and wavelength (391 to 397 nm) is observed at pH > 8.5, where the [Zn(H_−1_**L2**)]^+^ species prevails in solution. Being the mononuclear species the only significantly present in solution, the observed behavior could be attributed to a rearrangement of the species that affect the conjugation of the BPH moiety.

In the case of **L2** in the presence of a double amount of Zn(II) compared to the ligand (Figure 6c,d), the mononuclear species are prevalent in solution up to pH 7. Up to this pH value, a similar discussion as for **L2**: Zn(II) 1:1 molar ratio can be made also for the **L2**: Zn(II) 1:2 molar ratio. Starting from pH 7 the dinuclear species prevail in solution: the formation of [Zn_2_(H_−1_**L2**)]^3+^ induces a small increase of the fluorescence emission and a blue-shift of the emission wavelength from 390 to 386 nm. In this range of pH (7–9) a change of the absorption wavelength from 305 to 299 nm is also observed. All these phenomena could be attributed to the formation of the [Zn_2_(H_−1_**L2**)]^3+^ dinuclear species. The formation of the next [Zn_2_(H_−2_**L2**)]^2+^ species in the pH range 9–10 is accompanied by a small drop of both absorption and fluorescence emission, with a red-shift of the emission wavelength from 386 to 392 nm. The absorption wavelength keeps on blue shifting, reaching 297 nm. All these observations are related to the change of the protonation degree of BPH, that becomes fully deprotonated, along with the simultaneous coordination of each Zn(II) ion by one phenolate function of BPH, as already reported [45]. Finally, the formation of the neutral hydroxylated species [Zn_2_(H_−2_**L2**)(OH)_2_] at pH > 10 makes the fluorescence emission increasing again, suggesting again an increase in the BPH conjugation upon binding of OH^−^.

### 2.3. PMG and AMPA Binding by Zn(II) Complexes of ***L1*** and ***L2***

In the previous section we showed that **L1** and **L2** could be considered as useful chemosensors of H^+^ and Zn(II), since they are able to change their optical absorption and emission properties as a function of pH and in the presence of the metal cation. As for the Zn(II) complexes, most of them showed to be prone to add external anionic guests, such as OH^−^. Moreover, in the case of dinuclear complexes of **L2**, two Zn(II) centers are placed at short distance between each other, thus providing distinct anchoring sites for the simultaneous binding of multiple negatively charged residues of a given substrate. Altogether, these considerations make the metal complex species of **L1** and **L2**, hereinafter referred to as R systems (R1 = Zn(II) mononuclear complexes of **L1** and R2 = Zn(II) mono- and dinuclear complexes of **L2**), particularly attractive as metallo-receptors for anions. For this reason, and in view of the growing need for suitable tools to recognize the environmentally relevant glyphosate and AMPA, herein we decided to investigate the ability of the R metallo-receptors to bind such important analytes in aqueous solution, as well as their capability to detect their presence via fluorescence signaling.

#### 2.3.1. Potentiometric Measurements

**Figure 7:**
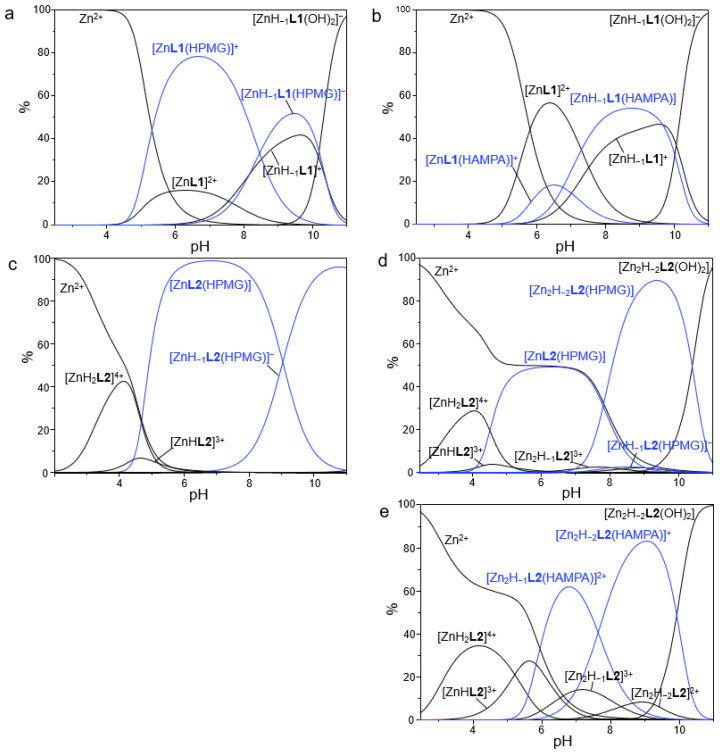
Distribution diagrams of the species formed in solution for the systems Zn(II)/**L1**/PMG (**a**), Zn(II)/**L1**/AMPA (**b**), Zn(II)/**L2**/PMG (**c**), 2Zn(II)/**L2**/PMG (**d**) and 2Zn(II)/**L2**/AMPA (**e**). [**L1**] = [**L2**] = [PMG] = [AMPA] = 1 × 10^−3^ M, [Zn^2+^] = 1 × 10^−3^ M (**a**–**c**) and 2 × 10^−3^ M (**d**,**e**), NMe_4_Cl 0.1 M, 298.1 ± 0.1 K.

The capacity of R metallo-receptors to bind PMG and AMPA was investigated through potentiometric measurements in NMe_4_Cl 0.1 M solution at 298.1 ± 0.1 K. R:A (A = PMG or AMPA) molar rations varying from 0.2:1 to 2:1 was employed, to ascertain the stoichiometries of the ternary adducts formed in solution. The resulting stability constants are summarized in Table 3, whereas the corresponding distribution diagrams of the species present in solutions are respectively shown in Figure 7.

A first analysis of data reveals that PMG and AMPA form stable adducts with both the mono- and dinuclear complexes of **L1** and **L2**, with the only exception of the mononuclear complexes of **L2**, for which no evidence of AMPA binding was found. Only AR adducts with 1:1 stoichiometry was observed under our experimental conditions, thus ruling out the coordination of multiple substrates to the same receptor unit but rather hinting at the simultaneous binding of a single guest to distinct anchoring sites of the host, likely through a bridge disposition (*vide infra*).

As it can be easily appreciated from Table 3, among the two substrates PMG led to the formation of the most stable adducts with the different forms of the two metallo-receptors. For example, the addition of HPMG^2−^ to [Zn_2_(H_−2_**L2**)]^2+^ occurs with a LogK value of 5.97, whereas the addition of HAMPA^−^ to the same species takes place with a LogK of 4.74; similar considerations can be made for **L1**.

The comparison between the two metallo-receptors unveils instead a higher affinity of PMG for **L2**, in both its mono- and dinuclear Zn(II) complex forms. This can be highlighted, for example, by LogK values of 6.97 and 3.54, respectively found for the addition of HPMG^2−^ to [Zn(H_−1_**L2**)]^+^ and [Zn(H_−1_**L1**)]^+^. Overall, the LogK values relative to the PMG coordination emerge to be comparable or up to 2 log units higher relative to the ones reported in literature for the binding of this substrate by analogous Zn(II)-polyamine receptors [56,57,58].

As shown in the distribution diagrams reported in Figure 7, the ternary adducts are formed at intermediate pH values, within 4 and 11, as expected due to the presence in this interval of pH of highly charged forms of both the metallo-receptors and the anionic substrates. In particular, the most relevant species formed by PMG with the two receptor systems emerged to be [Zn**L1**(HPMG)], [Zn(H_−1_**L1**)(HPMG)]^−^, [Zn**L2**(HPMG)] and [Zn_2_(H_−2_**L2**)(HPMG)] (Figure 7a,c,d), which are present in solution in a wide range of pH, from 4 to 11. On the other side, AMPA is mainly present in the adducts [Zn**L1**(HAMPA)]^+^, [Zn(H_−1_**L1**)(HAMPA)], [Zn_2_(H_−1_**L2**)(HAMPA)]^2+^ and [Zn_2_(H_−2_**L2**)(HAMPA)]^+^ (Figure 7b,e), which occur in a slightly narrower range of pH, from 5 to 11. For more alkaline pH values, the hydroxylated species of the metallo-receptors become to be predominant in solution.

**Figure 8:**
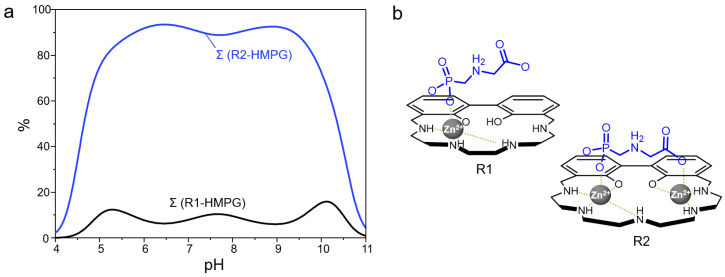
Selectivity diagrams showing the affinity of glyphosate (HPMG^2−^) with the metal complex species of **L1** and **L2** (R1 and R2, respectively) as a function of pH. Percentages were calculated with respect to ligand concentrations ([R1] = [R2] = [HPMG] = 1 × 10^−3^ M, R1 = ∑Zn(II)-mononuclear species of **L1** bound and R2 = ∑Zn(II)-mono- and dinuclear species of **L2** bound) (**a**). Models proposed for the coordination of HPMG^2−^ by the metallo-receptors [Zn(H_−1_**L1**)]^+^ and [Zn_2_(H_−2_**L2**)]^2+^, charges are omitted for clarity (**b**).

The superior binding properties of R2 towards PMG as compared to R1 can be better evidenced by the selectivity diagrams calculated for a competitive system containing PMG, R1 and R2 in equimolar amounts (R1 = ∑Zn(II)-mononuclear species of **L1** bound and R2 = ∑Zn(II)-mono- and dinuclear species of **L2** bound). Indeed, as shown in Figure 8, where are reported the overall percentages of the adducts formed by the different metallo-receptors with PMG (in its monoprotonated form HPMG^2−^) as a function of pH, this substrate is preferentially bound to R2 over a wide range of pH, from 4 to 11. In strict analogy with our previous study [48], this finding can be possibly rationalized by considering that, in the dinuclear complexes of **L2**, two coordinatively unsaturated Zn(II) ions can cooperate in the binding of PMG, with the latter being likely coordinated through its phosphate and carboxylate groups in a bridge disposition, with a Zn-O-C-C-N-C-P-O-Zn arrangement (Figure 8b). The simultaneous involvement of two distinct Zn(II) ions in R2 would be therefore clearly advantageous if compared to the anion coordination by R1, where only one metal-based anchoring site is available. Interestingly enough, the different binding abilities observed between the mononuclear complexes of the two ligands (Figure S5) could be tentatively explained by assuming that these metallo-receptors still display two potential anchoring sites, being represented by a coordinated Zn(II) ion on one side and by protonated nitrogen atom/s (-NH_2_^+^-) on the other side. In this view, the prolonged O-C-C-N-C-P-O scaffold of the guest would better match the larger Zn(II)-NH_2_^+^ distance in the 5-membered macrocycle of **L2**, compared to the smaller cavity (4-membered macrocycle) of **L1**, thus justifying the higher affinity of PMG towards the mononuclear complex species of **L2**. Accordingly, the lower affinity of both R1 and R2 towards AMPA would be the result of the worse fit between the smaller anionic bite of this guest (the O-donors are gathered on the same phosphate group) and the above proposed ditopic motif of the metallo-receptors.

#### 2.3.2. Fluorescence Measurements

**Figure 9:**
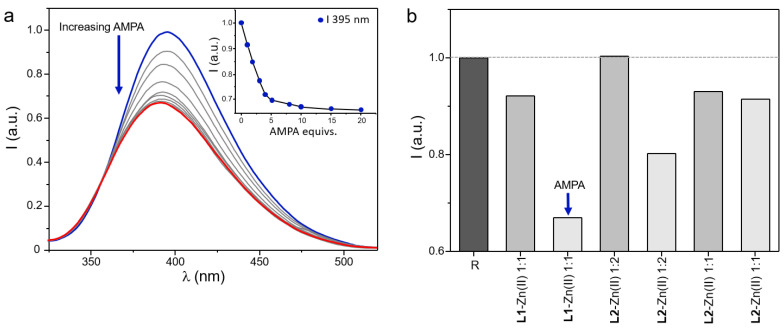
Fluorescence spectra of aqueous solutions containing **L1** and Zn(II) in 1:1 molar ratio at pH 9 and collected by adding increasing amounts of AMPA; in the inset is reported the variation of the fluorescence emission at 395 nm as a function of the concentration of the anion (**a**). Histogram showing the variations at the maximum fluorescence emission of metal complex species of **L1** and **L2** (R systems) in the presence of 5 equivs. of PMG (gray bars) and AMPA (light grey bars) (The blue color highlights the starting point of the fluorescence titration whereas the red line highlights the last measurement of the titration, registered in the presence of an excess of anionic species.) (**b**) ([R] = 5 µM, λ_ex_ = 270 nm). (The grey scales further highlight the data collected in the presence of the different anionic guests tested, whereas the dark grey bar evidences the reponse of the of metal complexed species of **L1** and **L2** (R systems) for comparison.)

Besides the analysis of the binding properties of the Zn(II) complexed forms of **L1** and **L2** towards PMG and AMPA, the ability of these metallo-receptors to undergo a variation of their fluorescence emission as a result of the coordination of the same anions was also evaluated. To this aim, aqueous solutions of R systems were added with increasing amounts of a selected anionic substrate and the resulting spectra were collected. For each system, measurements were performed at a fixed pH value, which was selected to allow the formation of a solution of the most stable adducts between the metallo-receptors and the anionic substrates. The coordinative selectivity highlighted by potentiometric data, which indicated the R2 systems as the most effective in the coordination of PMG, was not paralleled by the same optical trend. In fact, in general, the luminescent properties of R systems were only poorly affected by the coordination of the tested anions, with the only exception being represented by the adducts formed by R1 with AMPA. In this case, as shown in Figure 9a, the presence of increasing concentrations of AMPA in a solution at pH 9 of R1 determined a progressive quenching of the fluorescence emission of this system, resulting in ca. 35% decrease induced by a relatively small amount of analyte (5 equivalents). In all the other cases, the same amount of PMG/AMPA led to small variations of fluorescence emission that did not exceed 15% of the signal of the unbounded metallo-receptors (Figure 9b). Since a PET mechanism could not be usually invoked in the case of BPH-containing ligands, the observed quenching upon addition of AMPA to R1 could be rationalized in terms of coplanarity of the BPH group: a distortion of this moiety could be hypothesized in the binding of a non-well-fitting substrate such as AMPA, as discussed above. The lost in coplanarity would diminish the conjugation of BPH, and, finally, the fluorescence emission.

## 3. Conclusions

In this study, we explored the potential as metallo-receptors for glyphosate (PMG) and AMPA of the Zn(II) complexes of two polyamino-phenolic ligands, characterized by a fluorogenic biphenolic unit nicely placed into macrocyclic polyamine fragments of four (**L1**) and five (**L2**) nitrogen atom members.

Among the two ligands, **L2** was prepared by employing a novel synthetic approach, which consisted of a modification of the Richman-Atkins method. This allowed us to avoid the use of the toxic cadmium(II) ion, which was instead necessary for the previous template synthesis.

Following the study of the acid-base properties of **L1** and **L2**, their ability to bind Zn(II) in an aqueous solution was investigated by means of potentiometric, UV-Vis, and fluorescence measurements. Thanks to their peculiar macrocyclic design, these ligands allowed the formation of stable mono (**L1**) and both mono and dinuclear complexes (**L2**) with Zn(II), resulting in promising metallo-receptors for anionic targets.

The ability of the Zn(II) complexes of **L1** and **L2** (R1 and R2) to act as metallo-receptors for glyphosate and AMPA was inspected through potentiometric measurements. These studies highlighted that R1 and R2 strongly interact with the anionic substrates, with both receptors preferentially binding PMG over AMPA.

Comparative studies especially proved that PMG was preferentially coordinated by R2, also in competition with R1. This was assumed to be due to a ditopic motif for PMG coordination by R2, where the carboxylate and phosphate residues of the anionic guest are simultaneously coordinated to metal centers, in a bridge disposition among them.

Interestingly, R2 systems displayed a higher affinity for PMG over R1, even in their Zn(II) mononuclear forms. This led us to speculate that the ditopic coordination motif could be preserved in the mononuclear forms of **L2**, through the possible involvement, besides the metal, of protonate nitrogen atom/s (-NH_2_^+^-) of the polyamine framework.

Lastly, the ability of R systems to detect the presence of the selected analytes through fluorescence signaling in an aqueous solution was evaluated. In general, the luminescent properties of R1 and R2 were slightly affected by the coordination of the tested anions, with the only exception being represented by the adducts formed by R1 with AMPA.

In conclusion, this work further highlights the intriguing perspectives arising from the use of polyamino-phenolic ligands in the design of promising metallo-receptors for the recognition and sensing of elusive anions of great environmental relevance. It especially shows that slight differences in the structural architectures of this class of compounds may strongly affect not only their binding properties but even their behavior as fluorimetric chemosensors.

## 4. Materials and Methods

### 4.1. Synthesis

Ligands **L1** and **L2** were obtained following the synthetic procedure reported in Scheme 1. 3,3′-Bis(bromomethyl)-2,2′-dimethoxybiphenyl (**1**) [59,60,61], 1,4,7,10-tetrakis(*p*-tolylsulphonyl)-1,4,7,10-tetrazadecane (**2**) [62] and 1,4,7,10,13-pentakis(*p*-tolylsulphonyl)-1,4,7,10,13-pentaazatridecane (**3**) [63] were prepared as previously described. All other chemicals were purchased, using the highest quality commercially available. The solvents were RP grade, unless otherwise indicated.

#### 4.1.1. 23,24-Dimethoxy-3,6,9,12-tetrakis(4-methylbenzenesufonyl)-3,6,9,12-tetraazatricyclo[17.3.1.1(14,18)]eicosatetra-1(23),14,16,18(24),19,21-hexaene (**4**)

Over a period of 4 h, a solution of **1** (2.0 g, 5.0 mmol) in 100 mL of anhydrous acetonitrile was added to a refluxing suspension of **2** (3.8 g, 5.0 mmol) and K_2_CO_3_ (6.9 g, 50 mmol) in 250 mL of anhydrous acetonitrile, under nitrogen. The reaction mixture was refluxed for further 24 h. Subsequently, the mixture was cooled down to room temperature (R.T.) and the resulting suspension was concentrated under reduced pressure to one third of the initial volume, then poured into stirred cold water (1 dm^3^). The resulting white precipitate was filtered off, washed with cold water, dried under vacuum and purified by flash chromatography (hydrated alumina, dichloromethane/chloroform 70/30 *v*/*v*) obtaining **4** as a white solid (1.8 g, 36%).

^1^H NMR (CDCl_3_, 25 °C): δ = 2.41 (s, 6H), 2.48 (s, 6H), 2.92 (s, 6H), 2.97–3.52 (m, 12H), 3.94 (d, *J* = 14.3 Hz, 2H), 4.65 (d, *J* = 14.3 Hz, 2H), 7.15–7.23 (m, 4H), 7.30 (d, *J* = 8.1 Hz, 4H), 7.37 (d, *J* = 8.1 Hz, 4H), 7.53–7.62 (m, 2H), 7.70 (d, *J* = 8.3 Hz, 4H), 7.79 (d, *J* = 8.3 Hz, 4H) ppm. ^13^C NMR: δ = 21.5, 21.6, 47.3, 47.8, 48.9, 49.0, 61.2, 125.1, 127.3, 127.5, 129.8, 129.9, 130.5, 131.3, 132.0, 132.1, 135.2, 136.7, 143.5, 143.6, 155.7 ppm.

#### 4.1.2. 23,24-Dihydroxy-3,6,9,12-tetraazatricyclo[17.3.1.1(14,18)]eicosatetra-1(23),14,16,18(24),19,21-hexaene Tetrahydrochloride (**L1·4HCl**)

Ammonia (300 mL) was condensed in a suspension of **4** (1.5 g, 1.5 mmol) in diethyl ether (30 mL) and methanol (1 mL) and cooled down to −70 °C. Small pieces of lithium were carefully added to the mixture until the suspension turned blue. After thirty minutes, NH_4_Cl (12 g, 0.2 mol) was added. The white solid obtained after the evaporation of the solvent was treated with 3 mol dm^−3^ HCl (3 × 100 mL). The acidic solution was filtered and then evaporated to dryness, then the resulting solid was dissolved in the minimum amount of water and the solution made alkaline with concentrated NaOH. The liquid was extracted with CHCl_3_ (6 × 50 mL). The organic phase was dried over Na_2_SO_4_ and vacuum-evaporated to obtain a solid that was dissolved in ethanol and treated with 37% HCl/ethanol 1:1 *v*/*v* until complete precipitation of a white solid, which was filtered off to obtain **L1** as tetrahydrochloride salt (490 mg, 68%).

Anal. Calcd. for C_20_H_32_N_4_O_2_Cl_4_: C 47.82; H 6.42; N 11.15. Found: C 47.7; H 6.5; N 11.1. MS *m*/*z* (ESI): 357.5 (M + H^+^). ^1^H NMR (D_2_O, pH 2, 25 °C): δ = 3.18–3.57 (m, 12H), 4.39 (s, 4H), 7.14 (t, *J* = 7.6 Hz, 2H), 7.38 (d, *J* = 7.8 Hz, 2H), 7.43 (d, *J* = 7.6 Hz, 2H) ppm. ^13^C NMR: δ = 41.4, 43.0, 43.8, 46.0, 118.6, 122.4, 126.1, 132.5, 133.6, 152.5 ppm.

#### 4.1.3. 26,27-Dimethoxy-3,6,9,12,15-pentakis(4-methylbenzenesufonyl)-3,6,9,12,15-pentaazatricyclo[20.3.1.1(17,21)]eicosaepta-1(26),17,19,21(27),22,24-hexaene (**5**)

**5** was obtained following the same procedure used for **4** (**1** (1.9 g, 4.7 mmol); **3** (4.5 g, 4.7 mmol) and K_2_CO_3_ (6.5 g,47 mmol); **5** (1.2 g white solid, 21%)).

^1^H NMR (CDCl_3_, 25 °C): δ = 2.40 (s, 6H), 2.41 (s, 6H), 2.47 (s, 3H), 2.89 (s, 6H), 2.93–3.21 (m, 6H), 3.23–3.61 (m, 10H), 4.24 (d, *J* = 14.2 Hz, 2H), 4.57 (d, *J* = 14.4 Hz, 2H), 7.06–7.15 (m, 4H), 7.24–7.38 (m, 10H), 7.49–7.58 (m, 2H), 7.63 (d, *J* = 8.3 Hz, 2H), 7.73 (d, *J* = 8.3 Hz, 4H), 7.75 (d, *J* = 8.3 Hz, 4H) ppm. ^13^C NMR: δ = 21.4, 21.5, 21.6, 47.2, 48.2, 49.3, 49.5, 51.0, 60.8, 124.3, 127.4, 127.5, 127.6, 129.6, 129.7, 129.8, 129.9, 131.7, 131.8, 132.2, 135.1, 136.8, 143.2, 143.6, 143.9, 156.1 ppm.

#### 4.1.4. 26,27-Dihidroxy-3,6,9,12,15-pentaazatricyclo[20.3.1.1(17,21)]eicosaepta-1(26),17,19,21(27),22,24-hexaenepentahidrochloride (**L2·5HCl**)

**L2·5HCl** was obtained following the same procedure used for **L1·4HCl** (**5** (1.5 g, 1.2 mmol); **L2·5HCl** (490 mg white solid, 68%)).

Anal. Calcd. for C_22_H_38_N_5_O_2_Cl_5_: C 45.41; H 6.58; N 12.04. Found: C 45.4; H 6.5; N 12.1. MS *m*/*z* (ESI): 400.5 (M + H^+^). ^1^H NMR (D_2_O, pH 2, 25 °C): δ = 3.33–3.42 (m, 8H), 3.45–3.53 (m, 8H), 4.37 (s, 4H), 7.11 (t, *J* = 7.7 Hz, 2H), 7.34 (dd, *J* = 1.7, 7.6 Hz, 2H), 7.41 (dd, *J* = 1.5, 7.6 Hz, 2H) ppm. ^13^C NMR: δ = 42.4, 43.7, 44.2, 44.6, 47.3, 118.6, 122.0, 125.1, 132.4, 133.7, 152.4 ppm.

### 4.2. Potentiometric Measurements

The equilibrium constants for protonation, Zn(II)-complexation, glyphosate and AMPA binding by the Zn(II)-complexes of **L1** and **L2**, were determined by means of potentiometric (pH-metric) titrations in degassed 0.10 M NMe_4_Cl at 298.1 ± 0.1 K, employing equipment and procedures which have been already described [64,65,66].

Briefly, a combined glass electrode was calibrated as a hydrogen-ion concentration probe by titrating known amounts of HCl with CO_2_-free NaOH solutions, employing an Ag/AgCl electrode in saturated KCl as reference electrode. The equivalent point was determined by the Gran’s method [67], which afforded to obtain the standard potential E^o^ and the ionic product of water (pK_w_ = 13.83 ± 0.01 in our experimental conditions). All the employed solutions were prepared by using freshly boiled, doubly deionized water, saturated with anhydrous nitrogen prior to uses; NaOH solutions were standardized against carbonate free potassium hydrogen phthalate and stored under nitrogen atmosphere.

Measurements were performed by using a total ligand concentration of 1 × 10^−3^ M, in a range of pH within 2–11. For each system were performed at least three titration experiments, consisting of ca. 100 data points each. The relative equilibrium constants were determined from EMF data by using the program HYPERQUAD [68] while the distribution diagrams of the species present in the solution were obtained by the Hyss program [69].

### 4.3. Spectrophotometric and Fluorescence Measurements

Electronic UV-Vis absorption spectra were collected on a Perkin-Elmer Lambda 6 spectrophotometer and on a Varian Cary-100 spectrophotometer equipped with a temperature control unit, whereas fluorescence emission spectra were registered on a spectrofluorometer Horiba FluoroMax Plus and a Varian Cary-Eclipse spectrofluorimeter (spectra are uncorrected) by using an excitation wavelength of 288 nm. All measurements were performed at 298 ± 0.1 K.

## Figures and Tables

**Table 1:** Protonation constants (LogK) of **L1** and **L2** determined in NMe_4_Cl 0.1 M at 298 ± 0.1 K.

Reaction	LogK	
	L1	L2
H_−1_L^−^ + H^+^ = L	10.34 (9) ^1^	10.22 (8)
L + H^+^ = HL^+^	10.14 (6)	9.99 (8)
HL^+^ + H^+^ = H_2_L^2+^	6.89 (9)	8.70 (9)
H_2_L^2+^ + H^+^ = H_3_L^3+^	3.60 (9)	4.32 (8)
H_3_L^3+^ + H^+^ = H_4_L^4+^	2.28 (9)	3.47 (8)

^1^ Values in parentheses are standard deviations on the last significant figures.

**Table 2:** Stability constants of the Zn(II) complexes with **L1** and **L2** determined in NMe_4_Cl 0.1 M at 298 ± 0.1 K.

Reaction	LogK	
	L1	L2
H_−1_**L**^−^ + Zn^2+^ = [Zn(H_−1_**L**)]^+^	12.14 (9) ^1^	12.98 (9)
[Zn(H_−1_**L**)]^+^ + H^+^ = [Zn**L**]^2+^	7.54 (8)	7.89 (9)
**L** + Zn^2+^ = [Zn**L**]^2+^	9.54 (7)	10.88 (8)
[Zn**L**]^2+^ + H^+^ = [Zn(H**L**)]^3+^	-	7.39 (8)
[Zn(H**L**)]^3+^ + H^+^ = [Zn(H_2_L)]^4+^	-	5.21 (7)
[Zn(H_−1_**L**)]^+^ + OH^−^ = [Zn(H_−1_**L**)(OH)]	-	2.44 (8)
[Zn(H_−1_**L**)]^+^ + 2OH^−^ = [Zn(H_−1_**L**)(OH)_2_]^−^	7.32 (8)	-
H_−1_**L**^−^ + 2Zn^2+^ = [Zn_2_(H_−1_**L**)]^3+^	-	18.47 (8)
H_−1_**L**^−^ + 2Zn^2+^ = [Zn_2_(H_−2_**L**)^2+^ + H^+^	-	10.16 (8)
[Zn(H_−1_**L**)^]+^ + Zn^2+^ = [Zn_2_(H_−1_**L**)]^3+^	-	5.49 (8)
[Zn_2_(H_−2_**L**)]^2+^ + 2OH^−^ = [Zn_2_(H_−2_**L**)(OH)_2_]	-	9.16 (7)

^1^ Values in parentheses are standard deviations on the last significant figures.

**Table 3:** Logarithms of the equilibrium constants (LogK) for the coordination of Zn(II) complexes of **L1** and **L2** with glyphosate and AMPA determined by means of potentiometric measurements in 0.1 M NMe_4_Cl at 298 ± 0.1 K.

Reaction	L1
	LogK
[Zn(H_−1_**L1**)]^+^ + HPMG^2−^ = [Zn(H_−1_**L1**)(HPMG)]^−^	3.54 (9) ^1^
[Zn**L1**]^2+^ + HPMG^2−^ = [Zn**L1**(HPMG)]	4.46 (8)
[Zn(H_−1_**L1**)]^+^ + HAMPA^−^ = [Zn(H_−1_**L1**)(HAMPA)]	3.47 (9)
[Zn**L1**]^2+^ + HAMPA^−^ = [Zn**L1**(HAMPA)]^+^	2.74 (8)
	**L2**
	**LogK**
[Zn(H_−1_**L2**)]^+^ + HPMG^2−^ = [Zn(H_−1_**L2**)(HPMG)]^−^	6.97 (8)
[Zn**L2**]^2+^ + HPMG^2−^ = [Zn**L2**(HPMG)]	8.08 (9)
[Zn_2_(H_−2_**L2**)]^2+^ + HPMG^2−^ = [Zn_2_(H_−2_**L2**)(HPMG)]	5.97 (8)
[Zn_2_(H_−1_**L2**)]^3+^ + HAMPA^−^ = [Zn_2_(H_−1_**L2**)(HAMPA)]^2+^	4.25 (8)
[Zn_2_(H_−2_**L2**)]^2+^ + HAMPA^−^ = [Zn_2_(H_−2_**L2**)(HAMPA)]^+^	4.74 (7)

^1^ Values in parentheses are standard deviations on the last significant figures.

## Data Availability

The data presented in this study are available on request from the corresponding author.

## Supplementary Materials

The following supporting information can be downloaded at: https://www.mdpi.com/article/10.3390/molecules28052031/s1, Figure S1: (a) Absorption and (b) fluorescence spectra of **L1** at different pH values. [**L1**] = 1 × 10^−5^ M, λ_ex_ = 288, λ_em_ = 403 nm; Figure S2: Fluorescence spectra of **L1** + 1 equiv. of Zn(II) at different pH values (λ_ex_ 288 nm); Figure S3: Fluorescence spectra of **L2** + 1 equiv. of Zn(II) at different pH values (λ_ex_ 288 nm); Figure S4: Fluorescence spectra of **L2** + 2 equiv. of Zn(II) at different pH values (λ_ex_ 288 nm); Figure S5: Selectivity diagrams showing the affinity of glyphosate (HPMG^2−^) with the mononuclear Zn(II) complex species of **L1** and **L2** (R1 and R2, respectively) as a function of pH. Percentages were calculated with respect to ligand concentrations ([R1] = [R2] = [HPMG] = 1 × 10^−3^ M, R1 = ƩZn(II)-mononuclear species of **L1** bound and R2 = ƩZn(II)-mononuclear species of **L2** bound).
