# Supplementary material for: A New Family of Macrocyclic Polyamino Biphenolic Ligands: Acid-Base Study, Zn(II) Coordination and Glyphosate/AMPA Binding
Source: Molecules. 2023 Feb 21;28(5):2031. doi: 10.3390/molecules28052031 (PMC10003900; doi:10.3390/molecules28052031)
Supplement: Supplementary file 1 [file molecules-28-02031-s001.zip › molecules-2219453-supplementary.pdf]

A new family of macrocyclic polyamino biphenolic ligands: acid-base study, Zn(II) coordination and glyphosate/AMPA binding

Supplementary Material

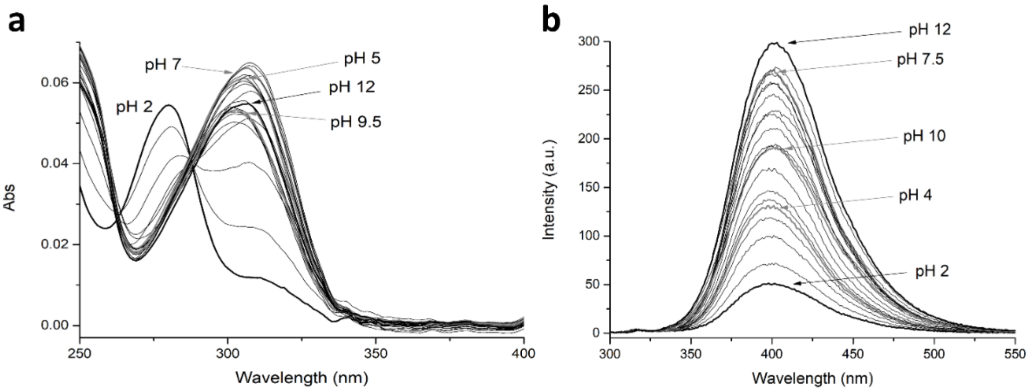

Figure S1. a) Absorption and b) fluorescence spectra of **L1** at different pH values. [**L1**] = 1 × 10^{-5} M, λ_{ex} = 288, λ_{em} = 403 nm.

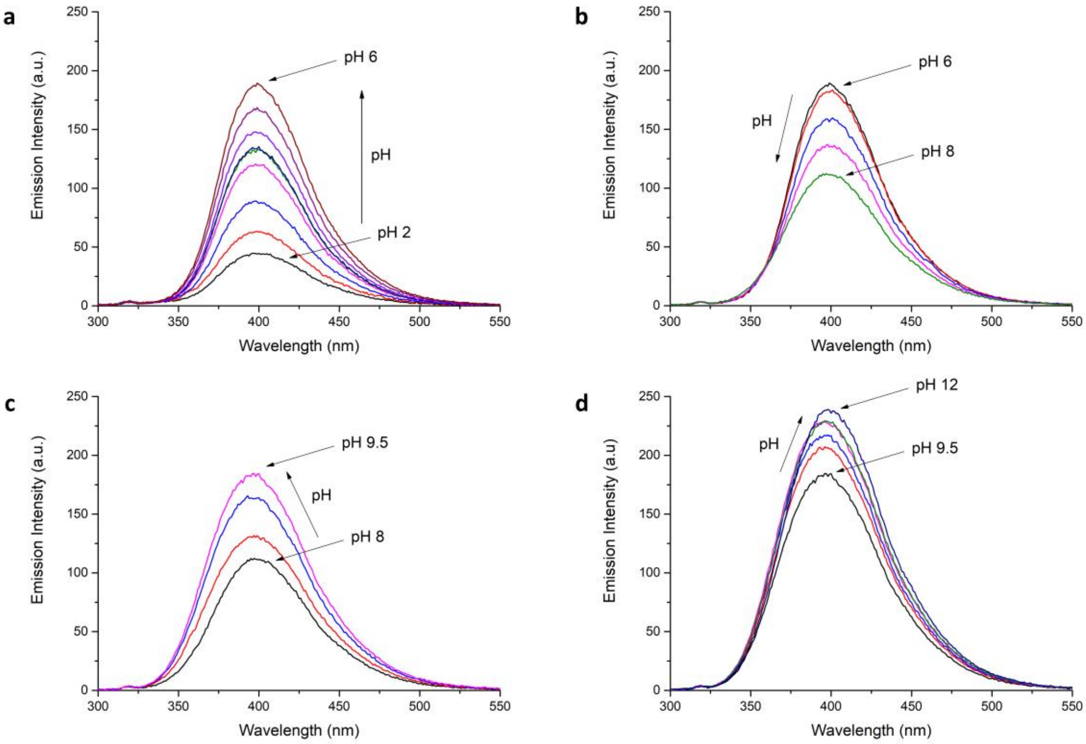

Figure S2. Fluorescence spectra of **L1** + 1 equiv. of Zn(II) at different pH values (λ_{ex} 288 nm).

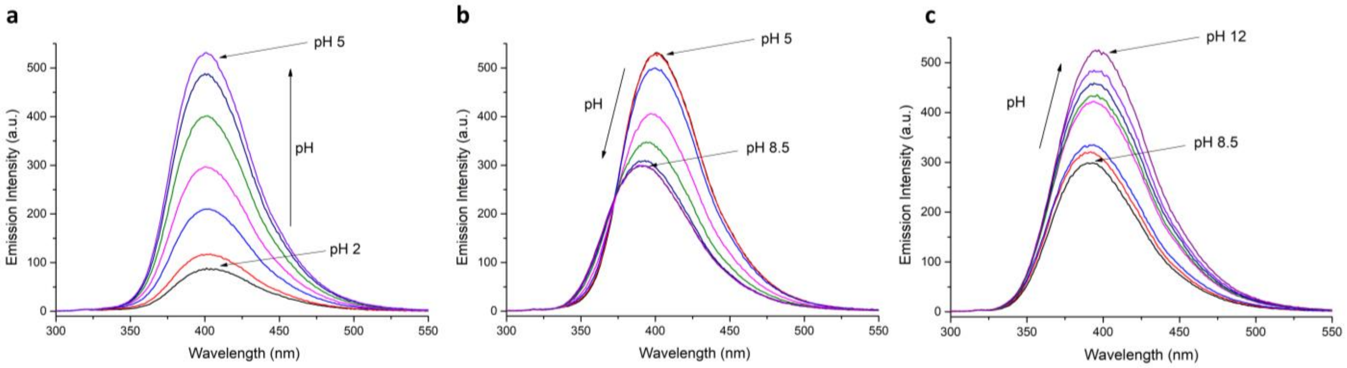

Figure S3. Fluorescence spectra of **L2** + 1 equiv. of Zn(II) at different pH values (λ_{ex} 288 nm).

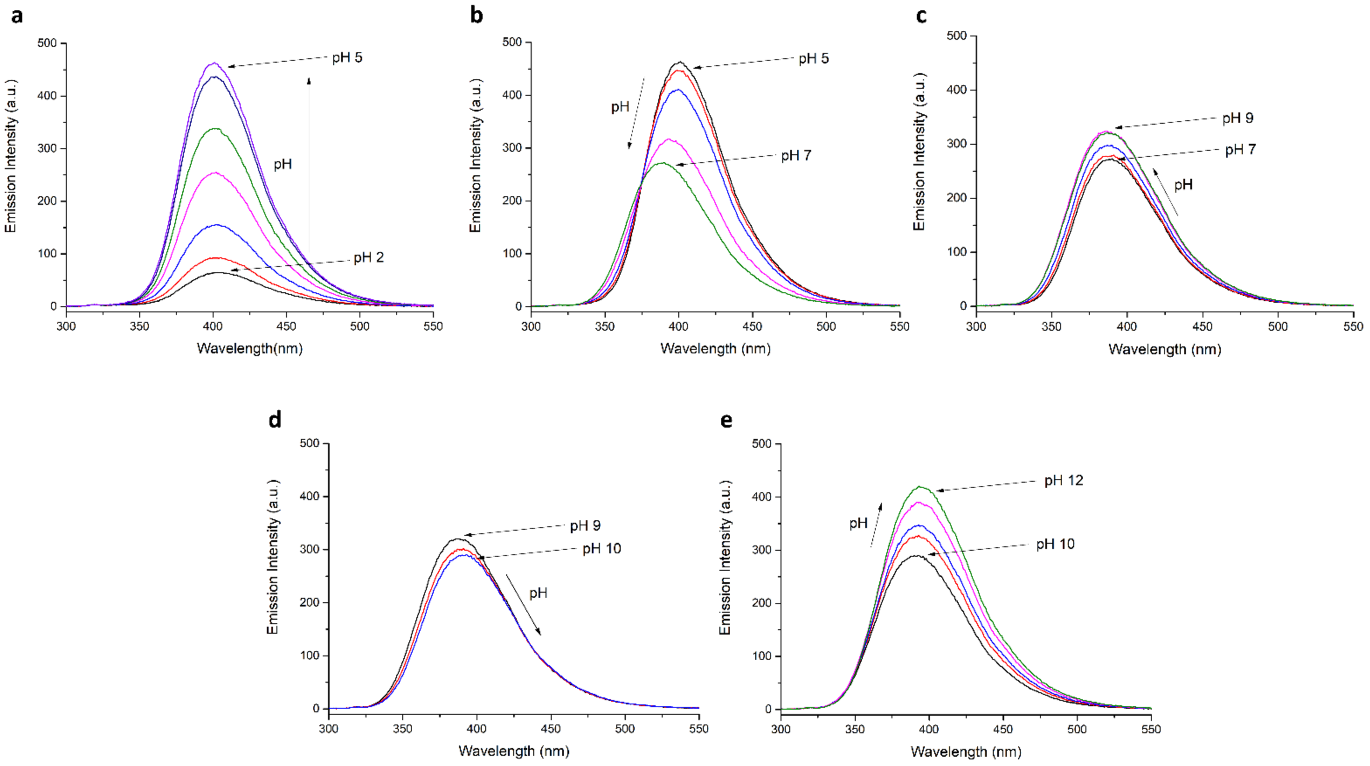

Figure S4. Fluorescence spectra of **L2** + 2 equiv. of Zn(II) at different pH values (λ_{ex} 288 nm).

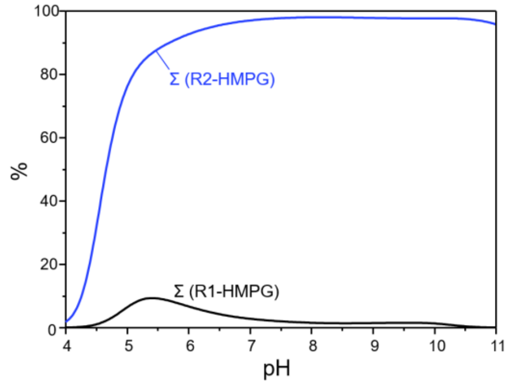

Figure S5. Selectivity diagrams showing the affinity of glyphosate ($HPMG^{2-}$) with the mononuclear Zn(II) complex species of **L1** and **L2** (R1 and R2, respectively) as a function of pH. Percentages were calculated with respect to ligand concentrations ([R1] = [R2] = [HPMG] = 1 x 10^{-3} M, R1 = ΣZn(II)-mononuclear species of **L1** bound and R2 = ΣZn(II)-mononuclear species of **L2** bound).
